# Fitness Effects of Food Resources on the Polyphagous Aphid Parasitoid, *Aphidius colemani* Viereck (Hymenoptera: Braconidae: Aphidiinae)

**DOI:** 10.1371/journal.pone.0147551

**Published:** 2016-01-25

**Authors:** Jennifer J. Charles, Timothy D. Paine

**Affiliations:** Department of Entomology, University of California Riverside, Riverside, California, United States of America; French National Institute for Agricultural Research (INRA), FRANCE

## Abstract

Conservation biological control involving the polyphagous aphid parasitoid, *Aphidius colemani* Viereck, may include provisioning resources from a variety of plant sources.

The fitness of adult *A*. *colemani* was enhanced with the provision of food resources such as floral nectar from a range of both native and introduced plant species and aphid honeydew under laboratory conditions. However, enhanced fitness appeared to be species specific rather than associated with the whether the plant was a native or an introduced species. Parasitoid survival and fecundity were enhanced significantly in response to the availability of floral nectar and honeydew compared to the response to available extrafloral nectar. These positive effects on the parasitoid’s reproductive activity can improve the effectiveness of conservation biological control in nursery production systems because of the abundance and diversity of floral resources within typical production areas. Additionally, surrounding areas of invasive weeds and native vegetation could serve as both floral resources and honeydew food resources for *A*. *colemani*.

## Introduction

More than 100 aphid species are considered pervasive pests that are difficult to control due to their high intrinsic rate of increase, varied reproductive strategies (parthenogenesis, telescoping generations, and viviparity), resistance to pesticides, role as virus vectors, and low aesthetic tolerance as crop contaminants [1, 2]. The melon aphid, *Aphis gossypii* Glover (Hemiptera: Aphididae), is one pestiferous species with a host range of more than 120 plant species representing 90 families [1, 2]. This aphid species is considered to be one of the most economically destructive aphids in the United States [3], and California nursery growers have reported aphids as one of the top ten pests in terms of pesticide use [4]. The melon aphid is a common pest of many containerized nursery crops including *Photinia x fraseri* Dress (Rosaceae), chrysanthemum, gardenia, and hibiscus.

Melon aphid population management on containerized plants is typically achieved through the means of chemical control. Large amounts of insecticides including organophosphates, pyrethroids, and carbamates are applied to achieve low aesthetic tolerance levels [4]. Increasing resistance to organophosphates and carbamates [5, 6, 7, 8] raises concerns for continued effective melon aphid population management solely through chemical control [9]. Consequently, melon aphid management in the containerized plant industry could shift to an increased reliance on biological control [9, 10].

The solitary endoparasitoid, *Aphidius colemani* Viereck (Hymenoptera: Braconidae: Aphidiinae), is a cosmopolitan species parasitizing over 41 aphid host species, including the melon aphid [11]. *A*. *colemani* has a similar intrinsic rate of increase as *A*. *gosypii* [12] and has the ability to discover and parasitize low density aphid populations [13]. Unlike many polyphagous aphid parasitoids [14], *A*. *colemani* readily accepts alternative aphid hosts potentially increasing its abundance in the field [15]. *A*. *colemani* has been considered an effective, but expensive, aphid control option for chrysanthemums and cucumbers in greenhouses [16, 17, 18], but the question of the parasitoid’s ability to reduce pest populations in the field has not been critically addressed.

In the California containerized nursery system, plants are grown in the open, often grouped into dense blocks of pots containing the same variety of plant in the same developmental stage. The blocks of one plant species will be interspersed with blocks of other species such that the growing area is a mosaic of blocks of hundreds of species of varieties of plants [19]. *A*. *colemani* is found in production areas and surrounding non-managed native vegetation as well as the invasive, weedy interface. This mixed-use agricultural landscape (the area in and surrounding the agricultural environment) provides a unique context to examine the potential effect that resource provisioning may have on improving the effectiveness of aphid biological control by *A*. *colemani*. While some groups of parasitoids obtain carbohydrates from host feeding [20], others can use hemipteran honeydew as a source of sugars [21]. The longevity of this parasitoid is enhanced when it has the opportunity to feed on floral nectar from a range of host plants [22]. Plants both within and around the production fields may serve as resources for aphid parasitoids providing nutrition and habitat while supporting the conservation of aphid parasitoids.

The practice of modifying the physical and biological properties of the agricultural environment to protect and enhance natural enemies was first defined as conservation biological control by van den Bosch and Telford [23], although the idea had been in use as early as 900 A.D. [24]. Enriching the agricultural environment with food resources such as floral (nectar and pollen), extrafloral (nectaries, exudates, and fruits), and insect products (honeydew and host feeding) is one method that may advance biological control by promoting natural enemy longevity and achieve an effective integrated approach to aphid management. Floral and extrafloral resources, along with insect products, are a necessary source of nutrition for adult parasitoids [25, 26, 27]. Some studies, especially laboratory assays, have shown that provisioning plant and insect-derived resources can increase longevity and fecundity [28, 29, 30, 31], parasitism rates [28, 32], and searching activity [33, 34]. In the field, plant-specific and aphid specific honeydews can influence parasitoid longevity [35, 36, 21, 37]; however, laboratory studies indicate that not all plant and insect products enhance natural enemy fitness. Factors such as floral morphology (corolla length and shape) [38], nectar components, and the relative ratios of those components in the nectar [39] significantly affect parasitoid accessibility and acceptance.

The objective of this study was to determine the fitness effects of naturally available and parasitoid accessible resources in order to evaluate the role resource provisioning may play in the conservation biological control of *A*. *gossypii* in California nursery systems. First, we evaluated weedy invasive, native, and ornamental floral resources for their potential to enhance *A*. *colemani* longevity and fecundity. Second, we investigated the effects of floral and honeydew resources on the longevity, fecundity, and sex ratio of *A*. *colemani* using resources collected from the containerized nursery plant, *P*. *x fraseri*, and its herbivore, *A*. *gossypii*.

## Materials and Methods

### Plant and insect colonies

All plant and insect colonies were maintained at the University of California, Riverside (UCR). Greenhouse grown colonies of *Cuburbito pepo* L. ‘Raven’ (Johnny’s Selected Seeds, Albion, ME) and *Brassica juncea* (L.) Czern. ‘Florida Broad Leaf’ (Ferry-Morse Seed Company, Fulton, KY) were cultivated in 0.92 L pots (industry standard 4” pots, Farrand Enterprises, Chino, CA) filled with UC Soil Mix I [40] and fertilized with *circa* 15 mL of Osmocote^®^ 18-6-12 (The Scotts Miracle-Gro Company, Marysville, OH). Plants and insect colonies were grown in natural light conditions at 25 ± 2°C with 25 ± 5% RH. Three week old plants of *C*. *pepo* ‘Raven’ were infested with *A*. *gossypii*. Another aphid, *Myzus persicae* (Sulzer), was grown on 3 week old *B*. *juncea* plants to serve as additional host material for the parasitoid. After 1 week, the two aphids on their respective host material were transferred to the greenhouse colony of the parasitoid, *A*. *colemani*, caged in a BugDorm2 (BioQuip^®^, Rancho Dominguez, CA). Parasitoids were held in the same conditions until mummies were removed for experiments. All treatments received a random assignment of parasitoids relative to the aphid host source. *A*. *gossypii* and the parasitoid were originally field-collected and maintained in the greenhouse for 2 months prior to experiments. *M*. *persicae* was acquired from a laboratory colony held at UCR.

### Floral resource effect on *A*. *colemani* fitness

#### Resource presentation

Floral resources from two invasive weed species, three native plant species, and three ornamental species associated with Southern California ornamental nurseries were evaluated for their potential as a food resource for the parasitoid *A*. *colemani*. Species selection was based on seasonal availability and represented 8 plant families. The three native species included *Encelia farinosa* A. Gray ex Torr. (Asteraceae), *Eriogonum fasciculatum* Benth. var. *foliolosum* (Nutt.) S. Stokes ex Abrams (Polygonaceae), and *Salvia apiana* Jeps. (Lamiaceae). The three ornamental species were *Lantana camara* L. (Verbenaceae), *Ligustrum japonicum* Thunb. (Oleaceae), and *P*. *x fraseri* (Rosaceae). The two invasive weed species were *Brassica nigra* (L.) Koch (Brassicaceae) and *Conium maculatum* L. (Apiaceae).

Inflorescences from mature, landscape individuals of each plant species in this experiment were bagged for 24 hours prior to removal. The white nylon sleeve bag (30.48 cm long with a 20.32 cm diameter) prevented other insects from removing resources. Inflorescences were cut, immediately placed in deionized water, and maintained in a sealed, ventilated container until the application of treatment. One inflorescence of *C*. *maculatum*, *E*. *fasciculatum*, *L*. *japonicum*, and *P*. *x fraseri* and two inflorescences of the remaining species were loaded into separate floral bouquet vases. The number of inflorescences used was based upon equalizing the floral count. The bouquet vase consisted of a single capped 40 dram plastic vial filled with deionized water. The inflorescences were held in place by inserting their stems through a standard hole-punch in the cap of the vial allowing the stem to pass into the deionized water. The thickness of the inflorescence stem(s) and the application of Parafilm M^®^ Laboratory Wrapping Film across the opening of the capped vial prevented contact between the parasitoid and the deionized water. A total of 11 treatments were applied; 8 floral, 1 honey-water (1:1 by weight) as a positive control, and 1 water (deionized water) as a negative control, plus a blank control. The honey-water and water treatments consisted solely of a 2 μL droplet of the resource applied to the bouquet vase. The honey was obtained from the University of California, Riverside bee colonies.

Mated female parasitoid individuals less than 24 hours old were caged in 1.82 L plastic cylinders with top (1, 11.43 cm diameter circle) and side (2, 5.72 cm diameter circles) ventilation covered with hardware cloth. The bottom of the cage was closed with a plastic 0.95 mL food container top (Smart & Final, Commerce, CA). One female *A*. *colemani* was placed inside each cage at the beginning of the experiment and maintained inside the same cage as the same fresh treatments were applied every 24 hours. Individual females were caged with treatments for 23 hours before a 1 hour aphid host-only exposure. Parasitoid longevity was recorded daily until the parasitoid died.

#### Host presentation

Aphid hosts were delivered to caged parasitoids via a 2–4 day old *C*. *pepo* ‘Raven’ plant grown in a 0.14 L pot (industry standard 2” pots from Farrand Enterprises, Chino, CA) using UC Soil Mix I [40] grown in natural light conditions at 25 ± 2°C with 25 ± 5% RH. All leaves were removed except for one true leaf that was infested with at least 100 first and second instars of *A*. *gossypii*, the preferred life stages for parasitization [41]. After the one hour exposure to the parasitoid, the aphid-infested plants were removed from the treatment cages and maintained under experiment environmental conditions for 8 days before mummies were removed and counted (parasitoid fecundity measurement). Environmental conditions during experiments were 25 ± 1°C with 15 ± 10% RH under inflorescent lighting with a L14:D10 photoperiod. The experiment was performed three times from April 2008 through May 2008 generating a range of 11 to 16 replicates per treatment. The number of treatments per experiment did not vary but escaped or lost replicates were not included in the analysis.

#### Statistical analysis

The data set was analyzed for treatment effects based on plant species using PROC GLM in SAS 9.3 [42]. Prior to analysis, the water and blank control treatments were removed from the data set due to lack of variance. The dependent variables, longevity and fecundity, were square root transformed to achieve normality. An analysis of variance (ANOVA) (*p* < 0.05, model adequacy based on residual analysis) comparing treatment, date, and their interaction was performed to verify there was no date effect across treatments. Based on a non-significant date effect, all experimental dates were pooled prior to the statistical analysis presented here. An ANOVA model and Tukey-Kramer’s test were constructed at a 0.05 significance level comparing plant species treatment at 9 levels (8 plant species plus a honey-water positive control). Data presented in this paper have been back transformed to the original values.

### Food resource use and *A*. *colemani* fitness

#### Collection and analyses of soluble carbohydrates

Two naturally occurring and parasitoid accessible resources, nectar and honeydew, were collected using the ornamental plant, *P*. *x fraseri*. Inflorescences of mature, landscape planted *P*. *x fraseri* were bagged with a white nylon sleeve bag to prevent insects from removing resources for 24 hours prior to nectar extraction using Drummond^®^ Short-Length Microcaps^®^ Micropipets (capillaries). Collections were taken during multiple dates in May 2007. Nectar was pooled from multiple inflorescences and multiple *P*. *x fraseri* individuals on each collection date. Prior to analysis and experimental use, all dates were combined. Honeydew, liquid aphid excrement, was collected from *A*. *gossypii* feeding on *P*. *x fraseri*. Parafilm M^®^ Laboratory Wrapping Film was wrapped around *P*. *x fraseri* leaves infested with *A*. *gossypii* and sealed to create an open-ended tube that rested on the leaf’s upper surface. The excreted honeydew accumulated and dried on the interior surface of the parafilm tube. After 24 hours, the parafilm was removed and laid open on the porcelain plate of a Fisher Scientifc^®^ Desiccator jar with 2 cm of deionized water in the bottom to create a saturated atmosphere in the jar for an additional 24 hours. Rehydrated honeydew was wiped off the parafilm with a Fisherbrand^®^ Spatula/Scraper. Collections occurred in February 2007 and honeydew was pooled from multiple leaves and multiple *P*. *x fraseri* individuals on each collection date. Prior to analysis and experimental use, all dates were combined. Both nectar and honeydew samples were stored at -16°C for less than 1 year. Three resources, honey-water, nectar, and honeydew, were analyzed for glucose, fructose, and sucrose by the University of California Agricultural and Natural Resources Analytical Lab (Davis, CA) using the quantitative method described by Johnson et al. [43] to determine if differences in sugar composition could be an explanatory factor in resource quality and fitness outcomes. Samples were extracted in water below the boiling point and subjected to analysis by HPLC.

#### Resource and host presentation

The fitness of *A*. *colemani* was assessed using three parameters, longevity, fecundity, and offspring sex ratio, in response to three naturally occurring and parasitoid accessible resources; extrafloral nectar, nectar, and honeydew. Two additional treatments, honey-water as a positive control and water as a negative control, along with a blank control were also assessed for a total of six treatments.

Mated female parasitoid individuals less than 24 hours old were kept in the same cages as previously described and given access to a resource treatment for 23 hours followed by a 1-hour aphid host exposure. Resource treatments consisted of a 2 μL droplet of resource applied to the cotyledon notch of a *C*. *pepo* ‘Raven’ plant, grown as previously described. All primary leaf growth was removed leaving only the cotyledons on each resource plant except for the extrafloral nectar treatment. The extrafloral nectar resource plant retained 1 true leaf containing extrafloral nectaries along with the cotyledons. This true leaf was the source of the resource. Resources were replaced every 24 hours to prevent depletion. *A*. *gossypii* hosts were delivered to the parasitoids for one hour as described in the previous floral resource experiment using *C*. *pepo*’ Raven” plants.

Aphid hosts were provided to each female every 24 hours and parasitoid longevity was recorded at that time. The exposed plants were removed from the parasitoid cages after the one hour exposure period and maintained under experimental conditions for 8 days before mummies were removed, counted, and allowed to eclose. Emerged females and males were counted in order to determine sex ratio and percent emergence. Experimental environmental conditions were the same as noted the previous trial. The experiment was performed 5 times from November 2007 through June 2008 generating a range of 12 to 35 replicates per treatment.

#### Statistical analysis

The data set was separately analyzed for treatment effects based on resource type using the GLM procedure in SAS 9.3 [42]. Prior to analysis, the water and blank control treatments were removed from the data set due to lack of variance. The dependent variables, longevity, fecundity (mummies and emerged individuals), percent emergence, and sex ratio, were square root transformed to achieve normality. An analysis of variance (ANOVA) (*p* < 0.05, model adequacy based on residual analysis) comparing treatment, date, and their interaction was performed to verify there was no date effect across treatments. Based on a non-significant date effect, all experimental dates were pooled prior to the analysis. An ANOVA model comparing resource treatment at 4 levels (extrafloral nectar, nectar, honeydew, and honey-water) was constructed at a 0.05 significance level with model adequacy based on residual analysis for each dependent variable. Treatment means were separated using the Tukey-Kramer’s test. Data presented in this paper have been back transformed to the original values.

### Hind tibia length measurements

Many parasitoid fitness characteristics including fecundity [44, 45] and longevity [46] are positively correlated with tibia length. Measurements of hind tibia length were taken on *A*. *colemani* individuals post-experiment to determine if parasitoid body size was related to fitness measurements. There was potential for body size variation as 2 aphid species, *A*. *gossypii* and *M*. *persciae*, were used to rear the parasitoid. The right metathorasic tibia was measured using an ocular micrometer inserted into a dissecting microscope. Measurements were taken at 800X magnification with a 0.02 mm resolution. One ANOVA model using the data set from the floral resource study was constructed at a 0.05 significance level with model adequacy based on residual analysis to compare hind tibia length across 9 treatment levels (8 floral species and 1 honey-water). A second ANOVA using the food resource data set was performed at a 0.05 significance level with model adequacy based on residual analysis with food resource treatments at 4 levels: extrafloral nectar, nectar, honeydew, and honey-water.

## Results

### Floral resource effect on *A*. *colemani* fitness

#### Floral species effect on longevity and fecundity

**Fig 1:**
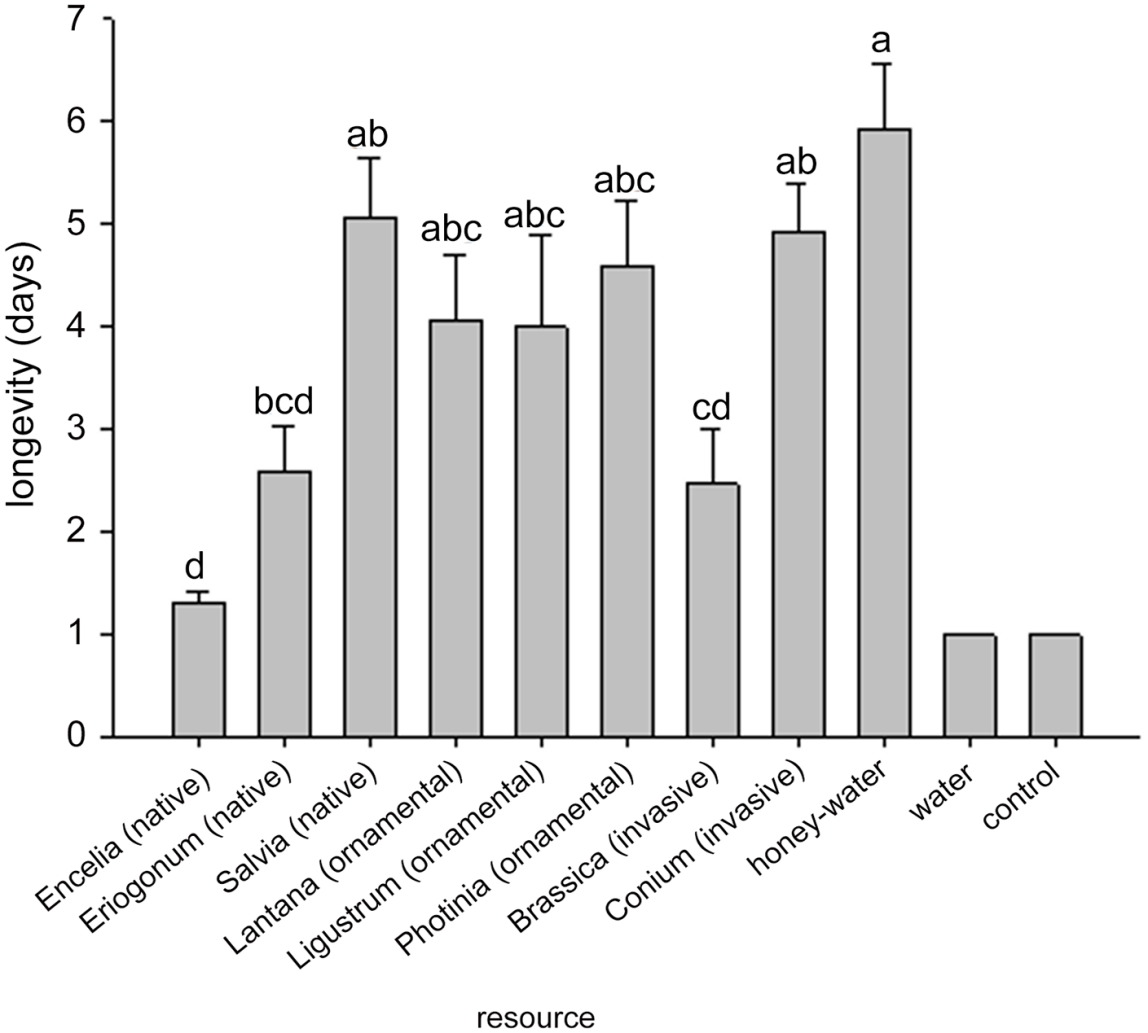
Survival of female *Aphidius colemani* on various food resources. Floral resources consisted of three ecological types, water, honey-water, and a blank control. Significant differences (*P* < 0.05) between resource treatments are indicated by different letters. The water and nothing treatment were not included in the analysis.

**Fig 2:**
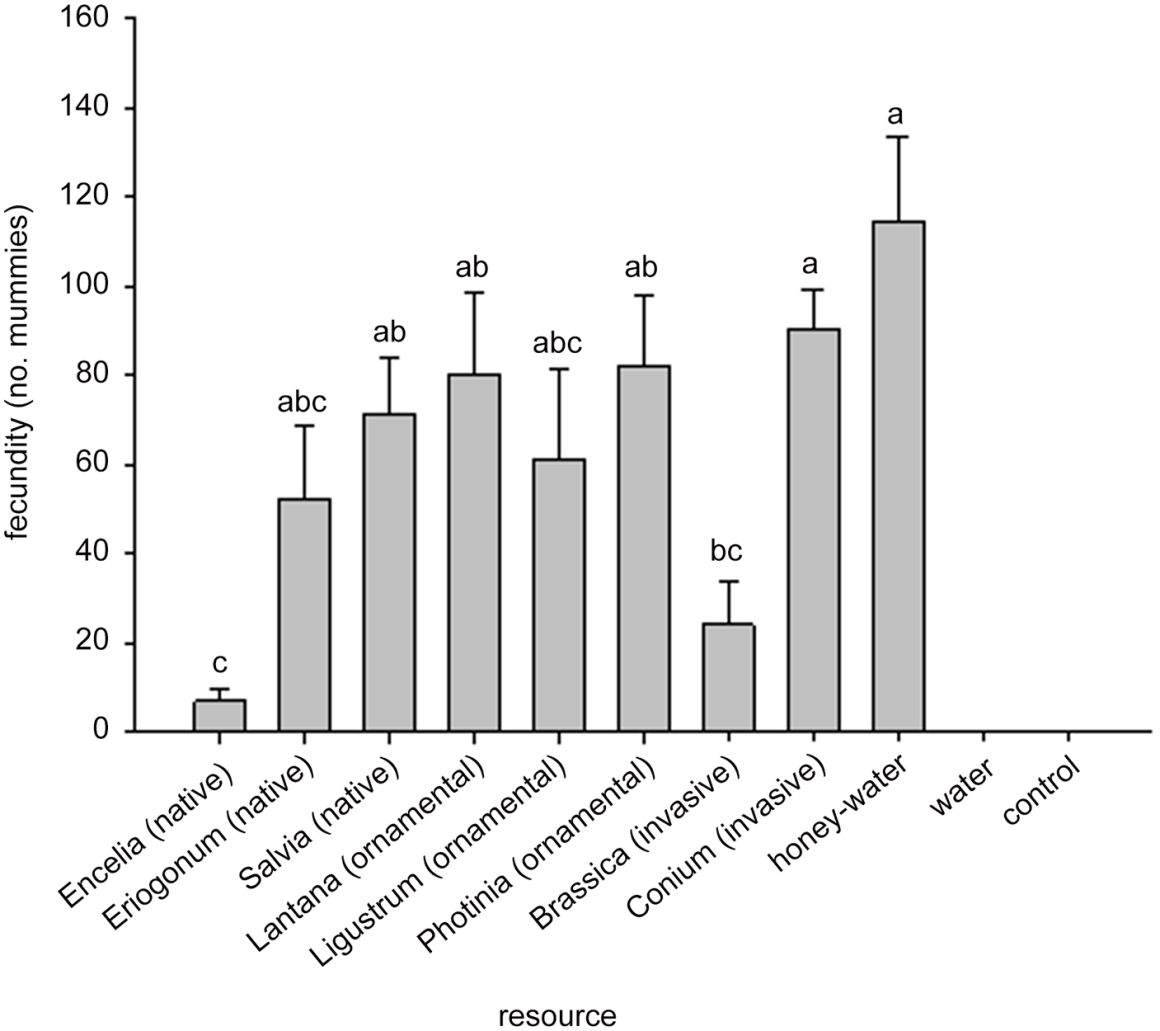
Fecundity of female *Aphidius colemani* on various floral food resources. Ecological classification indicated in parentheses. Significant differences (*P* < 0.05) between resource treatments are indicated by different letters. The water and blank control treatments were not included in the analysis.

A significant effect of floral species treatment was found for both fitness parameters, longevity (F_8,120_ = 7.26, *P* < 0.0001) and fecundity (F_8,120_ = 5.70, *P* < 0.0001). The water and blank control treatments were excluded from the analyses because of lack of variance. Longevity of *A*. *colemani* was not different for honey-water, *Salvia*, *Conium*, *Photinia*, *Lantana*, and *Ligustrum* treatments (Fig 1). Parasitoid longevity on the honey-water treatment was significantly greater than on *Eriogonum*, *Brassica*, *and Encelia* (Fig 1). Longevity on *Conium* was significantly greater than on *Brassica* and *Encelia* but not different from any of the other treatments. The fecundity of *A*. *colemani* was significantly higher for the honey-water *Conium* treatments than the *Encelia* and *Brassica* treatments, but not different from the *Eriogonum*, *Salvia*, *Lantana*, *Ligustrum*, and *Photinia* treatments (Fig 2).

### Food resource use and *A*. *colemani* fitness

There was a significant effect of resource treatment (extrafloral nectar, nectar, and honeydew) on *A*. *colemani* for four of the five fitness parameters; longevity (F _3,95_ = 9.62, *P* <0.0001), number of emerged offspring (F_3,95_ = 4.84, *P* = 0.0035), number of mummies (F_3,95_ = 4.11, *P* = 0.0086), and percent emergence (F_3,95_ = 2.76, *P* = 0.047). There was no statistical effect of resource treatment for sex ratio (F_3,95_ = 1.52, *P* = 0.21). The water and blank control treatments were not included in the analyses because all individuals died prior to presentation of the aphid oviposition hosts.

**Fig 3:**
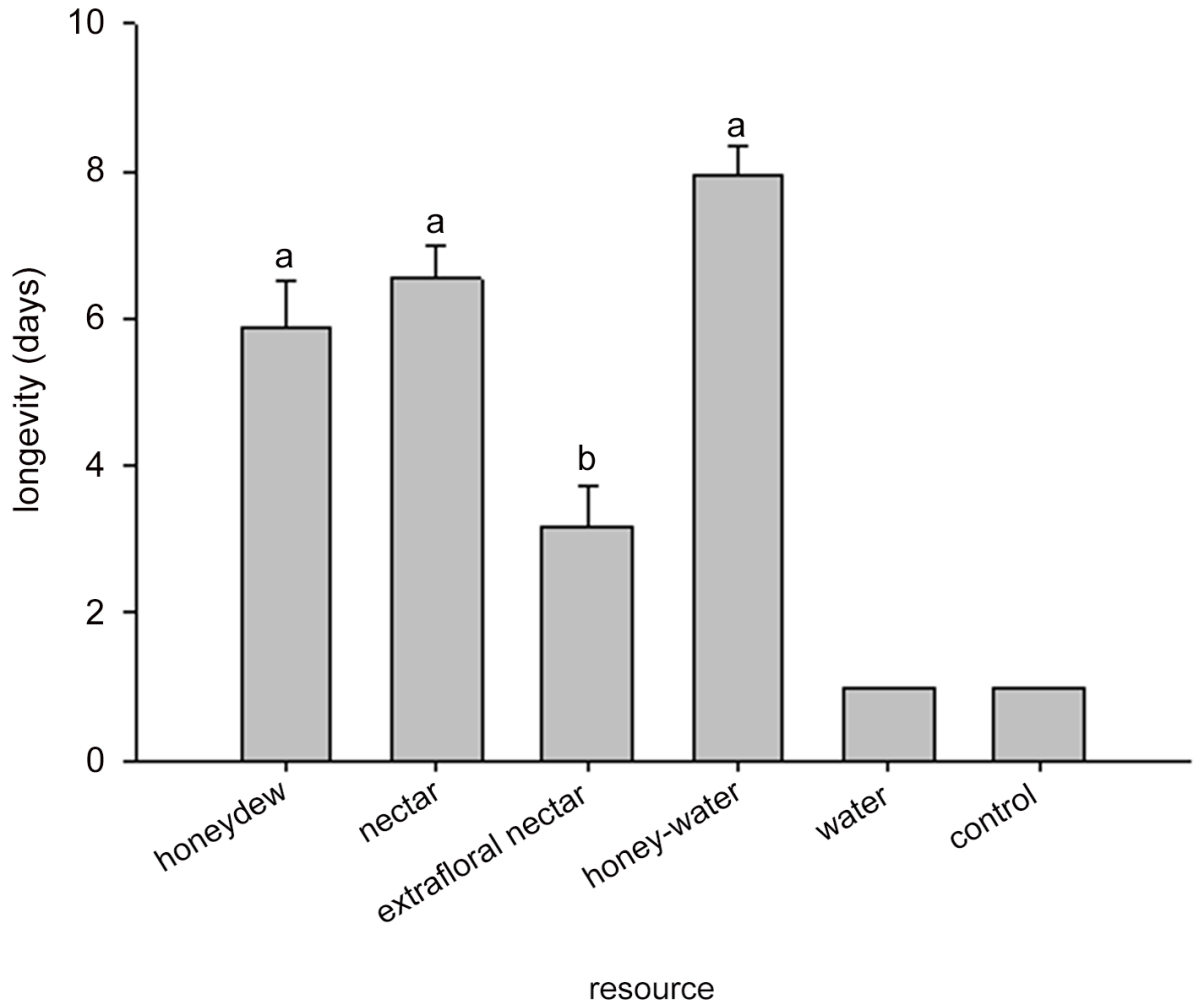
Survival of female *Aphidius colemani* on three naturally available and parasitoid accessible resources. Significant differences (*P* < 0.05) between resource treatments are indicated by different letters. The water and blank control treatments were not included in the analysis.

**Fig 4:**
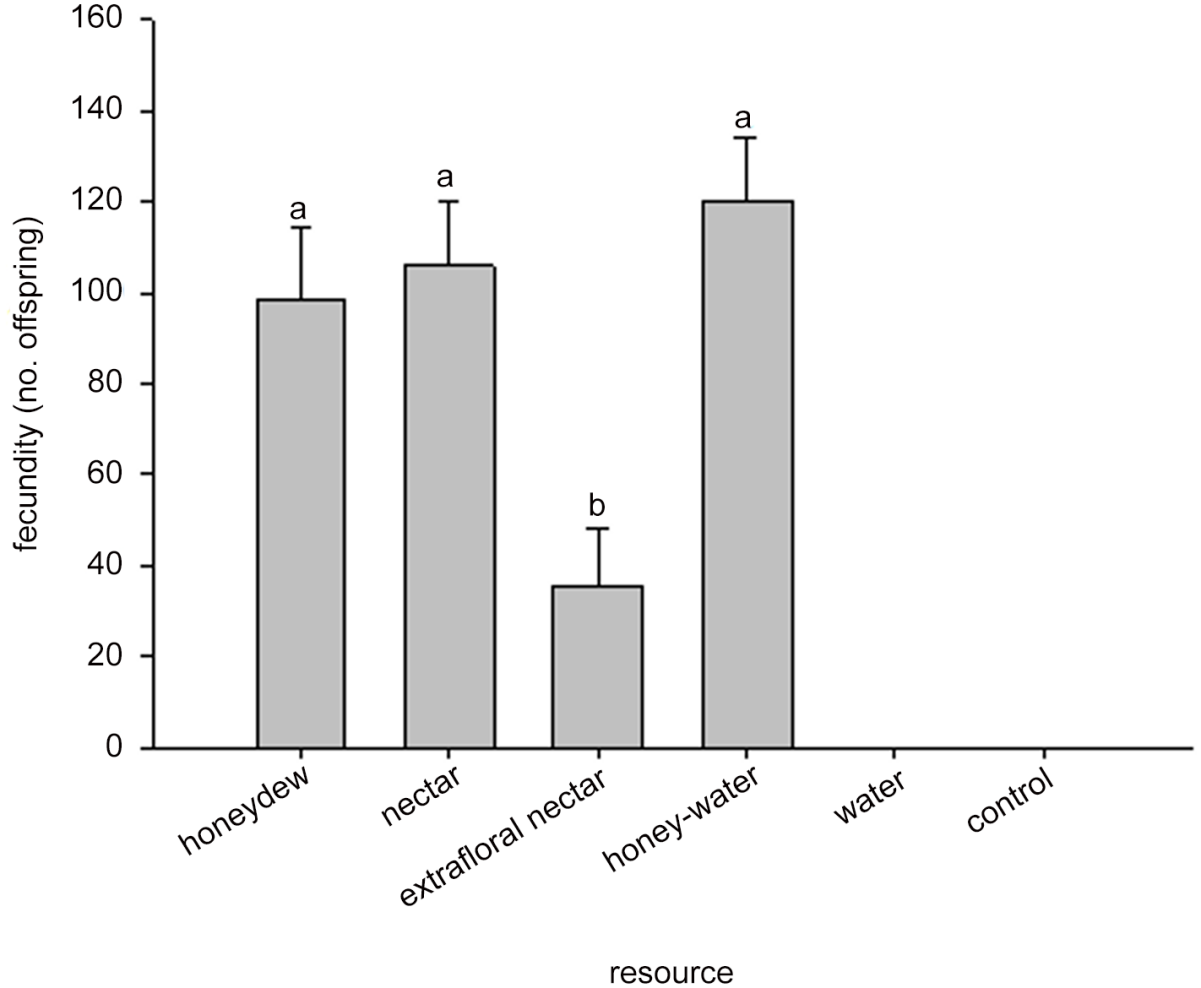
Fecundity of *Aphidius colemani* on three naturally available and parasitoid accessible resources. Significant differences (*P* < 0.05) between resource treatments are indicated by different letters. The water and blank control treatments were not included in the analysis.

There were no significant differences in longevity of *A*. *colemani* provided with honey-water, honeydew, or nectar as food resources (Fig 3). Adult wasp longevity on all three of these treatments was significantly longer than for wasps provided extrafloral nectar (Fig 3). Fecundity followed the same pattern as longevity; there were no significant differences in longevity of *A*. *colemani* provided with honey-water, honeydew, or nectar as food resources (Fig 4). Wasp fecundity was significantly lower when provided extrafloral nectar. The effect of food resource treatment on the percent emergence also followed the same pattern. There were no significant differences among the nectar (84.5 ± 8.5%), honey-water (82.1 ± 2.1%), and honeydew (65.3 ± 7.5%) treatments, but all these treatments were higher than the extrafloral nectar treatment (55.7 ± 8.8%).

### Hind tibia length measurements

There was no differences in length of the hind tibia of adults used across treatments in the floral resource trial (F_4,70_ = 0.55, *P* = 0.832) or the food resource trial (F_4,81_ = 1.06, *P* = 0.379). The results suggest that the source of the aphid hosts did not influence body size. Differences in fitness parameters observed here were not associated with differences in parasitoid body size.

### Soluble carbohydrates of food resources

All 3 sugar sources, honey-water, honeydew, and nectar, contained glucose, fructose, and sucrose (the only 3 sugars tested) (Table 1). Nectar, for the purposes of this study, contained the highest concentrations of all 3 sugars tested while honeydew contained the least (Table 1). The ratio of sucrose to its breakdown products, glucose and fructose, was 0.02 for honey-water, 0.06 for honeydew, and 0.08 for nectar (Table 1).

**Table 1:** Liquid sugar concentration (mg/L) of honey-water and 2 naturally available and parasitoid accessible resources.

Resource	Glucose	Fructose	Sucrose
honey-water	269000	151000	8150
honeydew	37100	6940	2720
nectar	312500	288500	47850

## Discussion

### Choosing floral resource candidates that are accessible, available, and maximize parasitoid fitness

The fitness benefits of accessible and available floral resources in agricultural systems have been supported by many previous studies [47, 48]. In this study, the longevity and fecundity of *A*. *colemani* was markedly improved with the provision of floral resources from 6 of the 8 plant species tested. These 6 species represent 6 different families, Apiaceae (*C*. *maculatum*), Rosaceae (*P*. *x fraseri*), Lamiaceae (*S*. *apiana*), Verbenaceae (*L*. *camara*), Oleaceae (*L*. *japonicum*), and Polygonaceae (*E*. *fasciculatum*). Species representing the Brassicaceae and Asteraceae were found to be nutritionally inferior. The four most prominent floral resource plants shown to enhance natural enemy fitness in the laboratory include species from the Apiaceae (*Coriandrum sativum* L.), Brassicaceae (*Lobularia maritima* (L.) Desv.), Hydorphyllaceae (*Phacelia tanacetifolia* Benth.), and Polygonaceae (*Fagopyrum esculentum* Moench) [49]. Although floral morphology is a key indicator in plant family identification [49] and in parasitoid accessibility [38], specific plant species must be evaluated for not only parasitoid accessibility but also for contributions to parasitoid fitness. Parasitoid accessibility and contribution to fitness, along with availability in the agricultural landscape, are likely critical factors in choosing appropriate candidate floral resources that support conservation biological control.

There was little obvious pattern in the origin of the most important fitness-enhancing plants in this study. Representatives of all three plant ecological classifications (invasive, ornamental, and native) available in the Southern California ornamental nursery and adjacent landscapes enhanced parasitoid fitness, while representatives of native and invasive species also ranked at the bottom. The landscape surrounding nursery production sites and its inherent floral resources offers an option to implement conservation habitat manipulation techniques that provide more than enhanced pest population control. However, choosing to provision with native floral resources instead of the typically utilized non-native floral resources may increase biodiversity and restore disrupted ecosystems adjacent to agricultural operations [50]. Native floral resources such as *S*. *apiana* not only increased the longevity and fecundity of *A*. *colemani* in this study but also may support additional ecosystem services that benefit both the agricultural environment and its surrounding ecosystems.

### Fitness enhancement through resource provisioning

Agricultural landscapes are typically depauperate of resources necessary for parasitoids to exert any significant population control over their pest hosts [51]. Provisioning supplemental resources including floral, extrafloral, and insect products has been one of the core efforts to conserve natural enemies and improve biological control in agriculture systems [52]. Floral resources have been the primary focus in this effort while extrafloral resources and insect products like honeydew have been less studied [52]. Supplying accessible floral resources not only attracts parasitoids to the landscape [53, 54], but also concentrates their presence which potentially increases control of pest populations [55, 56] while simultaneously boosting fitness parameters like longevity and fecundity [48, 57]. Suggestions about the mechanisms of honeydew’s impact on biological control are similar to those for floral resources but empirical data across parasitoid families has been lacking. The case supporting provision of extrafloral resources is also inadequate. Floral resources have been viewed as the superior sugar source [58, 39] and extrafloral resources have been demonstrated as suitable [59, 60, 61] and unsuitable [62], while honeydew has been generalized as an inferior resource for subsidizing natural enemies [63]. Alternatively, some soft scale honeydews may function as a higher quality resource [64].

Comparative analysis of the reproductive characteristics of *A*. *colemani* presented in this study examined three naturally available and parasitoid accessible sugar sources, floral, extrafloral, and honeydew along with honey-water. The longevity and fecundity of *A*. *colemani* were greatly improved when honey-water, nectar, and honeydew were provided as compared with water and the control but there appears to be little difference between these food resources. Extrafloral nectar was also different from the water treatment and the control but was not comparable to honey-water, nectar, and honeydew as longevity and fecundity were less than half of the other resource treatments. These results do not support the hypothesis that honeydew is an inferior sugar source [63].

Although honeydew provisioned in this study had the least amount of glucose, fructose, and sucrose as compared with nectar and honey-water, there were no differences among the three resources in enhancing parasitoid. An increasing number of studies are beginning to address the sugar profile of provisioned resources. A study of *Cotesia glomerata* (L.) (Hymenoptera: Braconidae) found that provisioning glucose (18%), fructose (18%), and sucrose (34%) were equally successful at extending the parasitoid’s lifespan as compared with water [39]. Hogervorst et al. [35] compared sucrose (68%) to various aphid honeydews from potato and wheat and reported no difference in the longevity of *Aphidius ervi* Haliday (Hymentoptera: Braconidae: Aphidiinae) when fed on potato honeydew generated by *M*. *persicae*. The sugar profile of *M*. *persciae* potato honeydew consisted of 2.2% glucose, 12.8% fructose, and 9.6% sucrose [35]. Honeydew from *A*. *gossypii* used in this experiment was more concentrated for glucose (1.68 times) but had much lower concentrations of both fructose (18.45 times) and sucrose (35.33 times) than *M*. *persicae*’s potato honeydew. The combined glucose, fructose, and sucrose sugar concentration of honeydew from *A*. *gossypii* feeding on *C*. *pepo* was approximately 19% of the concentration of the same three sugars in honeydew from *M*. *persicae* feeding on potato. This simple comparison of three studies demonstrates the potential variation in the sugar profiles of provisioned parasitoid food resources. Not only may this variation in sugar content and concentration explain differences in parasitoid fitness, but honeydews contain oligosaccharides that have been suggested to lower the nutritional quality [58, 39]. As the examinations of specific sugars and nutritional quality of provisioned resources proliferates, attention to sugar profiles, concentrations, and component ratios may help explain underlying mechanisms that boost parasitoid fitness which may ultimately contribute to the control of a pest population.

The containerized nursery production systems in California may include hundreds of different plant species or varieties arranged in blocks of pots set in high density spatial arrangements. Each block is typically uniform by plant type and phenological stage, but the diversity of plants among blocks in the same area may be very high. The result is a concentrated mosaic of both bloom and diversity of available nectar resources in a localized area. If insecticide applications are reduced in a shift to reliance on biological control, then high levels of plant and phenological diversity should also result in an increase in the number of suitable host plants and available hemipteran honeydew. In addition, the production areas are often adjacent to either urban landscapes with a wide diversity of native and introduced plant resources or wildlands with native plants with bloom periods more closely adapted to local environmental conditions. Because of the virtually continuous bloom, it is likely that floral nectar will be available to the parasitoids for much of the year. Plant diversity in the adjacent urban areas would supplement the nursery sources of both nectar and honeydew for nutrition of parasitoids and provide a buffer in the event that the nursery sources were temporarily unavailable.

## Conclusions

The fitness of *A*. *colemani* was enhanced with the provision of food resources such as floral nectar and aphid honeydew under laboratory conditions. The expectation is that these positive effects on the parasitoid’s reproductive activity can be translated into pest population control in the field. The ornamental nursery system offers an abundance of floral resource opportunities without the need to lose production space. Additionally, surrounding areas of invasive and native vegetation could serve as both floral resources and honeydew food resources for *A*. *colemani*. In order to demonstrate the real influence of provisioning food resources to *A*. *colmeani* on its aphid host, field trials assessing the populations of both *A*. *colemani* and *A*. *gossypii* are necessary. Consideration should also be given to the temporal and spatial fit with crop production schemes, the non-target impacts of provisioning (e.g., stimulating unintended pest outbreaks by providing alternative food sources for herbivores), and the economics of tactic implementation.
